# STGATE: Spatial-temporal graph attention network with a transformer encoder for EEG-based emotion recognition

**DOI:** 10.3389/fnhum.2023.1169949

**Published:** 2023-04-13

**Authors:** Jingcong Li, Weijian Pan, Haiyun Huang, Jiahui Pan, Fei Wang

**Affiliations:** School of Software, South China Normal University, Guangzhou, China

**Keywords:** EEG-based emotion classification, EEG, deep learning, graph neural network, transformer encoder

## Abstract

Electroencephalogram (EEG) is a crucial and widely utilized technique in neuroscience research. In this paper, we introduce a novel graph neural network called the spatial-temporal graph attention network with a transformer encoder (STGATE) to learn graph representations of emotion EEG signals and improve emotion recognition performance. In STGATE, a transformer-encoder is applied for capturing time-frequency features which are fed into a spatial-temporal graph attention for emotion classification. Using a dynamic adjacency matrix, the proposed STGATE adaptively learns intrinsic connections between different EEG channels. To evaluate the cross-subject emotion recognition performance, leave-one-subject-out experiments are carried out on three public emotion recognition datasets, i.e., SEED, SEED-IV, and DREAMER. The proposed STGATE model achieved a state-of-the-art EEG-based emotion recognition performance accuracy of 90.37% in SEED, 76.43% in SEED-IV, and 76.35% in DREAMER dataset, respectively. The experiments demonstrated the effectiveness of the proposed STGATE model for cross-subject EEG emotion recognition and its potential for graph-based neuroscience research.

## 1. Introduction

Emotion is a generalization of subjective human experience and behavior. Emotions affect our perceptions and attitudes dramatically and play an essential role in human-computer interaction (HCI) (Jerritta et al., 2011). Emotions have a significant impact on our evaluation, attitudes, behavior, decisions, cognition, learning, perception, and understanding (Brosch et al., 2013). Moreover, emotions can act as a motivational mechanism that enhances users' attention, interest, and motivation, thereby promoting their learning and cognition (Tyng et al., 2017). In the context of human-computer interaction (HCI), emotions can serve as a valuable feedback mechanism that increases user satisfaction, engagement, and loyalty (Brave and Nass, 2007; Jeon, 2017).

As a crucial and fundamental research area of affective computing and neuroscience, emotion recognition has attracted great attention from the academy and enterprise fields in recent years (Cambria et al., 2017; Torres et al., 2020). Emotion recognition technology can generally be categorized into two major categories (Shu et al., 2018). The first category involves non-physiological signals such as facial expressions, speech, gestures, and posture (Schuller et al., 2003; Anderson and McOwan, 2006; Castellano et al., 2008). The second category is based on physiological signals such as electroencephalogram (EEG), electrocardiogram (ECG), electromyography (EMG), skin temperature (SKT), and others (Egger et al., 2019). Physiological-based emotion recognition is considered more reliable as it is difficult for individuals to deliberately control their physiological signals. Among the physiological signals, EEG signals are widely used in neural engineering and brain-computer interfaces (BCIs) research due to their high temporal resolution, non-invasiveness, and low cost (Craik et al., 2019). Emotional states are closely related to neural activity produced by the central nervous system (Torres et al., 2020). This neural activity can be directly measured using EEG devices, making EEG-based emotion recognition increasingly popular in various fields, such as education, health, entertainment (Xu et al., 2018; Suhaimi et al., 2020; Abdel-Hamid, 2023; Moontaha et al., 2023).

A major problem with recognizing emotions is that emotions should be defined and accessed quantitatively. There are two different models used to define emotions: the discrete model and the dimensional model (Shu et al., 2018). According to the discrete model, emotions are divided into several basic categories, such as sadness, fear, disgust, surprise, happiness, and anger. These emotions can form more complex emotion categories through a certain combination of patterns (Peter and Herbon, 2006; Van den Broek, 2013). The dimensional emotion model maps emotional states into the points on a certain coordinate system. Different emotional states are distributed in different positions in the coordinate system, and the distance between positions reflects the difference between different emotional states (Wioleta, 2013; Poria et al., 2017; He et al., 2020). Different from discrete emotion models, the dimensional emotion model is continuous and has the advantages of a wide range of emotions and the ability to describe the evolution of emotions.

In recent decades, EEG-based emotion recognition has attracted much attention from researchers (Jerritta et al., 2011). A typical recognition process of emotional EEG usually consists of two parts: EEG feature extraction and emotion classification (Alarcao and Fonseca, 2017). EEG is a highly dynamic and nonlinear signal with a large amount and redundancy of data. Thus, feature extraction is an important step in emotion evaluation because high-resolution features are essential for effective pattern recognition (He et al., 2020). EEG features can be mainly divided into time-domain features, frequency-domain features, and time-frequency features (Jenke et al., 2014; Stancin et al., 2021; Huang et al., 2022). One of the widely used methods of frequency domain feature analysis of EEG signals is to decompose EEG signals into several frequency bands, including delta (1–3 Hz), theta (4–7 Hz), alpha (8–13 Hz), beta (14–30 Hz), and gamma (>31 Hz) (Aftanas et al., 2004; Davidson, 2004; Li and Lu, 2009). EEG features can be extracted from each band. The common time domain features include statistical features (Liu and Sourina, 2014) and Hjorth features (Hjorth, 1970). Commonly used frequency domain features include power spectral density (PSD) (Thammasan et al., 2016), differential entropy (DE) (Shi et al., 2013), and rational asymmetry (RASM) (Zheng et al., 2017). The common time-frequency domain features include wavelet features (Akin, 2002), short-time Fourier transform (Kıymık et al., 2005), and Hilbert-Huang transform (Hadjidimitriou and Hadjileontiadis, 2012).

One of the most successful methods for recognizing emotions based on EEG signals is Deep Neural Networks (DNN) (Zhang et al., 2020; Ozdemir et al., 2021). The Convolutional Neural Networks (CNN) method was proven to be a powerful tool to model structured data in many applications, ranging from image classification and video processing to speech recognition and natural language understanding (Gu et al., 2018). However, EEG signals can be considered non-Euclidean data in order to extract the relationship of different brain regions, and Convolutional Neural Networks (CNN) may not be effective in capturing the hidden patterns of non-Euclidean data (Micheloyannis et al., 2006). In recent years, Graph Neural Networks (GNNs) have been developed rapidly and offer a potential solution to extract correlation features among EEG channels in emotion recognition tasks (Wu et al., 2020). In the graph representation of emotional EEG signals, each EEG channel corresponds to a vertex node, and the connections between vertex nodes correspond to edges in the graph, making it suitable for encoding the correlation among the brain regions in the multichannel EEG signal (Jia et al., 2020). However, constructing a better graph representation of EEG signals for emotion recognition problems remains challenging as the spatial position, which must be predetermined before building the EEG emotion recognition model, is different from the functional connections among EEG channels (Song et al., 2018).

To leverage both spatial relationships and time-frequency information, many researchers have extended graph neural networks by spatial-temporal attention. Spatial temporal attention is a mechanism that captures the dynamic relationship between spatial and temporal dimensions in data. It consists of two kinds of attention: spatial attention, which focuses on the relevant regions or nodes in space; and temporal attention, which focuses on the time steps in time dimension. Sartipi et al. proposed the novel spatial-temporal attention neural network (STANN) to extract discriminative spatial and temporal features of EEG signals by a parallel structure of the multi-column convolutional neural network and attention-based bidirectional long-short term memory (Sartipi et al., 2021). Li X. et al. (2021) proposed a model called attention-based spatial=temporal graphic long short-term memory (ASTG-LSTM), in which a specific spatial-temporal attention embedded into the model to improve the invariance ability against the emotional intensity fluctuation. Liu et al. (2022) proposed a spatial-temporal attention to explore the relationship between emotion and spatial-temporal EEG features. Therefore, it is reasonable to consider incorporating spatial-temporal attention to improve classification accuracy.

In this paper, we propose a novel model, STGATE, which combines a transformer learning block (TLB) and a Spatial-temporal Graph Attention (STGAT) mechanism. TLB utilizes 2D convolutional layers and a transformer encoder to extract time-frequency information, while the STGAT utilizes both spatial and temporal attention mechanisms to learn connections between brain regions and temporal information, respectively. Our approach treats EEG signals as graph data and incorporates them into graph neural networks to capture correlations between EEG channels. Unlike the GNN methods, the adjacency matrix learned by STGATE can provide a better graph representation because it is adaptively updated by spatial attention during the training process. The main contributions of this paper can be summarized as follows:

- This paper proposes a novel spatial-temporal graph attention network with a transformer encoder (termed STGATE) for EEG-based emotion recognition.
- STGATE utilizes a transformer learning block and spatial-temporal graph attention. This allows it to capture electrode-level time-frequency representations. It also helps STGATE learn the emotional brain activities within and among different brain functional areas.
- STGATE achieved state-of-the-art performance with a cross-subject accuracy of 90.37% in SEED, 76.43% in SEED-IV, and 77.44% and 75.26% in the valence and arousal dimensions of the DREAMER dataset, respectively. Extensive ablation studies and analysis experiments were conducted to validate the efficiency of the proposed STGATE.

Remainder of this paper is organized as follows. The proposed STGATE method is presented in Section 3. The datasets and experiment settings are presented in Section 4. In Section 5, numerical emotion recognition experiments on the SEED, SEED-IV, and DREAMER datasets are carried out. In addition, the performance of the current methods and the proposed methods are presented and compared. Some discussions and analyzes of the proposed model are presented in Section 5. The conclusions of this paper are given in Section 6.

## 2. Related work

### 2.1. Emotion recognition

Emotion recognition is crucial for research in affective computing and neuroscience. Many studies on EEG-based emotion recognition focus on feature engineering or deep learning. Long short-term memory (LSTM) has been utilized to learn features from raw EEG signals and has achieved higher average accuracy than traditional techniques (Alhagry et al., 2017). A deep adaptation network has also been used to eliminate individual differences in EEG signals for effective model implementation (Li et al., 2018).

However, the inter-channel correlation of EEG signals for emotion recognition is critical. Song et al. (2018) proposed a novel dynamic graph convolutional neural network to dynamically learn the intrinsic relationship between different channels. To capture both local and global relations among different EEG channels, Zhong et al. proposed a regularized graph neural network (RGNN) for EEG-based emotion recognition, which models the inter-channel relations in EEG signals *via* an adjacency matrix (Zhong et al., 2020). A graph convolutional broad network was designed to explore the deeper-level information of graph-structured data and achieved high performance in EEG-based emotion recognition (Zhang et al., 2019). Li et al. proposed a Multi-Domain Adaptive Graph Convolutional Network (MD-AGCN), fusing the knowledge of both the frequency domain and the temporal domain to fully utilize the complementary information of EEG signals (Li R. et al., 2021) designed a model called ST-GCLSTM, which utilizes spatial attention to modify adjacency matrices to adaptively learn the intrinsic connection among different EEG channels (Feng et al., 2022).

Various methods and classifiers have been proposed and applied to the problem of EEG-based emotion recognition. To improve the accuracy of emotion recognition, this paper proposes STGATE, a model that extracts time-frequency and spatial features from EEG signals.

### 2.2. Graph attention network

According to previous studies, graph convolutional neural networks are divided into spectral and spatial methods (Chen et al., 2020). The spectral method uses the convolution theorem to map the signal to the spectral space, which overcomes the non-Euclidean data missing translation invariance feature. The spatial method operates directly on the graph data and achieves the convolution effect by aggregating the information of neighboring nodes.

Graph attention networks (GATs) are a kind of network based on an attention mechanism to classify graph-structured data, which belongs to the spatial method of graph convolutional neural network (Veličković et al., 2017). The basic idea is to calculate the hidden representation of each graph node in the graph data by aggregating the information of neighboring points using the self-attention strategy and to define the information fusion using the attention mechanism function. Unlike other graph networks, GAT calculates the association weights by the feature representations of the nodes instead of calculating the weights based on the information of the edges. The input to a graph attention network is a series of feature vectors of nodes, which can be expressed as $H=\left\{{\vec{h}}_{1},{\vec{h}}_{2},\ldots ,{\vec{h}}_{N}\right\},{\vec{h}}_{i}\in {\mathbb{R}}^{N\times F}$, where N is the number of vertices, and F represents feature dimensions. The graph attention network uses a self-attentive mechanism to compute the attention coefficients of the input feature vectors and normalize them as follows:


(1) $$\begin{array}{l} e_{ij}=a\left(W{\vec{h}}_{i},W{\vec{h}}_{j}\right) \end{array}$$

(2) $$\alpha_{ij}={\mathrm{Softmax}}_{j}\left(e_{ij}\right)=\frac{\exp\left(e_{ij}\right)}{\sum_{k\in N_{i}}\exp(e_{ik})}$$

where *e*_*ij*_ represents attention weights between node *i* and node *j*, and *a*_*ij*_ is the normalized attention weight, indicating the importance of node *i* to node *j*, $\vec{h}$ is the eigenvector; ***W*** is the weight matrix in (1, 2). The attention weights and expressions can be represented as follows:


(3) $$\alpha_{ij}=\frac{\exp\left(\text{LeakyReLU}\left({\vec{a}}^{T}\left[W{\vec{h}}_{i}∥W{\vec{h}}_{j}\right]\right)\right)}{\sum_{k\in N_{i}}\exp\left(\text{LeakyReLU}\left({\vec{a}}^{T}\left[W{\vec{h}}_{i}∥W{\vec{h}}_{k}\right]\right)\right)}$$

where || denotes the concatenation operation, *N*_*i*_ denotes the set of neighboring nodes of the *i* th node, ${\vec{\text{a}}}^{T}$ represents the transpose of the attention weight vector, and LeakyReLU denotes the nonlinear activation function in Equation (3). To make the network more informative, the graph attention network uses a multi-head mechanism that makes each head capture different information. The information from multiple heads is fused through a linear layer, and the attention coefficients are combined with the corresponding feature vectors to compute the final output features of each node.


(4) $${\vec{h}}_{i}^{\prime }=\sigma \left(\frac{1}{K}\sum_{k=1}^{K}\sum_{j\in N_{i}}\alpha_{ij}^{k}W^{k}{\vec{h}}_{j}\right)$$

where ***W*** is the weight matrix of the linear layer, σ is the nonlinear activation function, and ${\vec{h}}_{i}^{\prime }$ is the final output vector of the graph attention network in Equation (4).

The graph attention network assigns different weights to the nodes (EEG channels) through the attention mechanism, which effectively improves the representational capability of the network. At the same time, the graph attention network operates very efficiently with a computational complexity of *O*(|*V*|*FF*′+|*E*|*F*′), where *F* is the dimension of the input vector, |*V*| is the number of nodes, and |*E*| is the number of edges.

## 3. Methods

**Figure 1:**
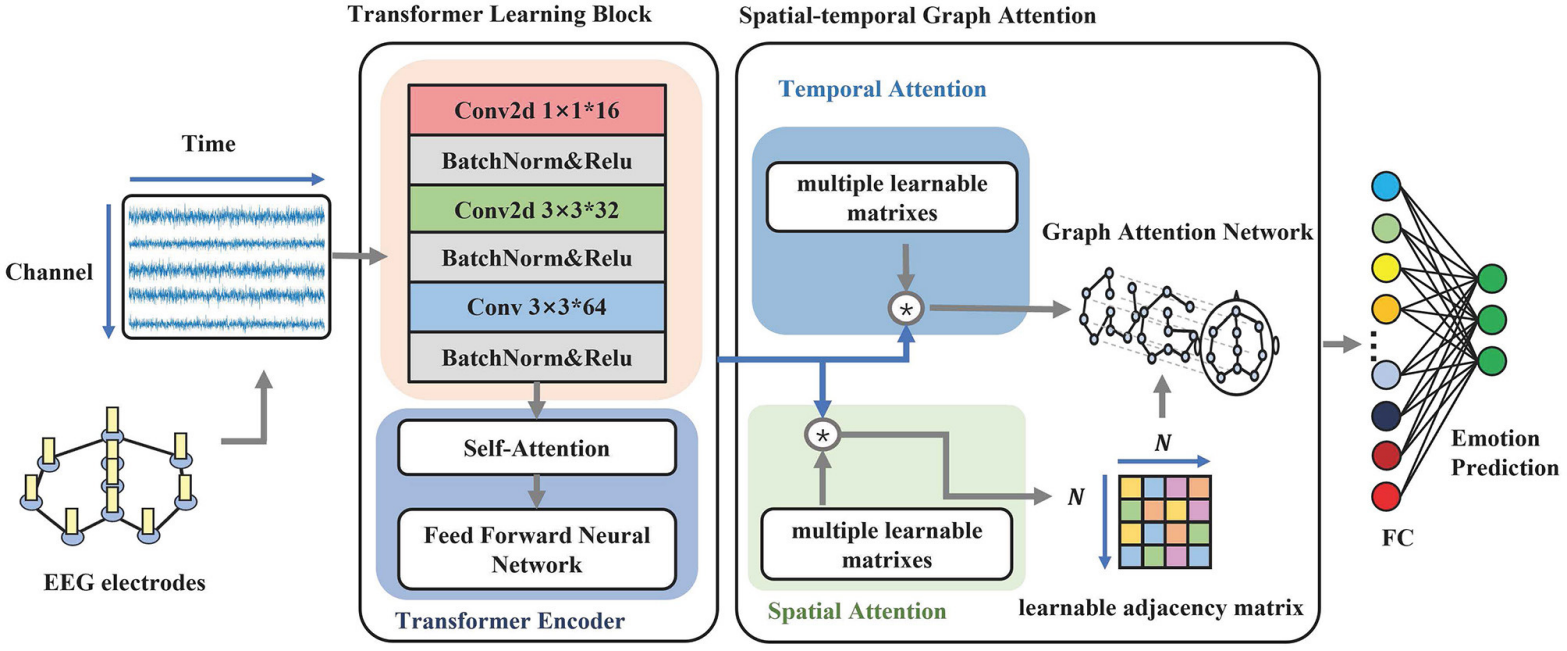
The overall structure of STGATE. The model contains two main modules, Transformer Learning Block and Spatial-Temporal Graph Attention. The first module aims to learn time-frequency representations. The second module focuses on building dynamic graph representations by using an attention mechanism.

As shown in Figure 1, the architecture for our proposed model consists of two modules. The upper module is an electrode-level learning block for extracting time-frequency information. The bottom module is a dynamic graph convolution for the correlation of EEG channels by constructing the adjacency matrix during the training and testing processes.

### 3.1. Transformer learning block

Figure 1 illustrates the transformer learning block (TLB), which aims to learn electrode-level time-frequency representations from EEG signals. TLB comprises two main components. The first component is a stack of multi-kernel convolutions that downsample five frequency bands and extract multi-scale features. Previous studies have reported that network connections in the high gamma band are denser among different emotional states, such as happiness, neutrality, and sadness, compared to other frequency bands (Yang et al., 2020). Similarly, Newson and Thiagarajan (2019) found that emotional disorders are more related to higher frequencies, including the alpha, beta, and gamma bands. Therefore, emotional states are more relevant to the alpha (8–13 Hz), beta (14–30 Hz), and gamma (>31 Hz) frequency bands (Ding et al., 2020). To better make use of all informative frequency bands, the kernels of the convolution layers are set to 1, 3, and 3. The 3*3 convolution layers are adopted followed by the 1*1 convolution layer, which aims to add network nonlinear features. Transformer-based methods have achieved great success in many areas (Raganato and Tiedemann, 2018; Liu et al., 2021). The multi-head self-attention and parallel inputting have superior abilities to capture long-range dependencies. The positional embedding learns the positional information of the sequence. To enhance the long-range dependencies captured in EEG, the second component uses a transformer encoder to map the EEG sequence to a new encoded sequence that contains more temporal information to enhance the long-range dependency capturing ability in EEG.

### 3.2. Graph representation

Usually, EEG signals are measured by placing the electrodes on the corresponding locations in the human brain scalp, and the brain electrodes measure voltage changes generated by neural activity in the cerebral cortex (Subha et al., 2010). The distribution position of the brain electrodes is defined by some standards, such as the international 10/20 system. The distribution position of the brain electrodes is fixed and regular, so the EEG signal channels can be considered classical non-Euclidean structured data (Micheloyannis et al., 2006), which are well suited to be represented by graphical data.


(5) G=(V,ℰ)

(6) $$V=\left\{v_{i}|i=1,\ldots ,N\right\}$$

(7) $$ℰ=\left\{e_{ij}|v_{i},v_{j}\in V\right\}$$

(8) $$A=\left\{a_{ij}\right\}$$

A segment of EEG signals collected by a brain electrode can be considered as a node of the graph. Therefore, we regard multi-channel EEG signals as a graph. G denotes a graph, V denotes the set of vertices in graph G, and E represents the set of edges in Equations (5–7). *N* is the number of brain electrodes in Equation (6). In the graph representation of EEG signals, a node *v*_*i*_ is usually used to represent an EEG electrode, while an edge *e*_*ij*_ represents the correlation between nodes *v*_*i*_ and *v*_*j*_. *A* is the adjacency matrix of graph G. *a*_*ij*_ represents the strength of the correlation of nodes *v*_*i*_ and *v*_*j*_ in Equation (8). The adjacency matrix ***A*** is a learnable matrix and can be dynamically modified during the training process. Generally, we model the EEG signal as an undirected graph and use this undirected graph as the input to the adaptive graph module. The initial set of edges of the undirected graph obtained from the above modeling is determined by the kNN algorithm, which computes graph edges to the nearest k points.

### 3.3. Spatial-temporal graph attention

Neural activity in different brain regions has an intrinsic correlation during the emotional experience. EEG signals recorded by brain electrodes can also reflect some intrinsic correlation in different brain regions. Therefore, we proposed spatial-temporal graph attention (STGAT) to capture correlations between EEG electrodes in the spatial domain and temporal EEG information in the temporal domain. Specifically, STGAT dynamically learns the adjacency matrix *A* through a spatial attention mechanism during the training process and uses temporal attention to further learn the temporal information in EEG.

Spatial attention can be implemented with the following formula:


(9) $$\begin{array}{l} S=V\cdot \sigma \left(W_{1}X_{h}W_{2}+b_{s}\right) \end{array}$$

(10) $$\begin{array}{l} A=\frac{S-E\left[S\right]}{\sqrt{\mathrm{Var}\left[S\right]}} \end{array}$$

where ***S***∈ℝ^*B*×*N*×*N*^ is a weight matrix, which represents the importance of edges. ***A*** represents the dynamic adjacency matrix. $X_{h}\in {\mathbb{R}}^{B\times N\times C\times T_{r}}$ is the input of the block. *B* is the batch size. *N* is the number of vertices of the input data. *C* represents the 2D convolution channels. *T*_*r*_ denotes the length of the temporal dimension. $V,b_{s}\in {\mathbb{R}}^{N\times N}$, $W_{1}\in {\mathbb{R}}^{T_{r}}$, and $W_{2}\in {\mathbb{R}}^{C\times N}$ are learnable parameters, and σ denotes the Tanh activation function. We adopt batch normalization to reduce internal covariate shifts and accelerate training (Santurkar et al., 2018). *E*[·] and *Var*[·] denote the mini-batch mean and mini-batch variance of ***S***. The value of an element ***S*_*ij*_** indicates the strength of the connection between node *i* and node *j*. We use the spatial attention matrix ***A*** as the adjacency matrix so that the adjacency matrix can be dynamically constructed by the corresponding input features. To obtain better representations of EEG signals, we adopt a Top-K algorithm to maintain the 10 edges with the highest weight and discard the others. The Top-K operation is applied as follows:


(11) $$\left\{ \begin{array}{l} \text{ for }i=1,2,\ldots ,N\\ \text{ index }=\mathrm{argtopk}(A[i,:])\\ A[i,\bar{\text{ index }}]=0 \end{array}\right.$$

where the *argtopk*(·) is a function to obtain the index of the top-k largest values of each vector in Equation (11). The use of a dynamic adjacency matrix in EEG emotion recognition has contributed to the ability to dynamically learn the intrinsic relationship between different EEG channels, which can reflect the brain connectivity patterns associated with different emotional states. Moreover, the dynamic adjacency matrix can adapt to different subjects, thereby improving the cross-subject generalization ability of EEG emotion recognition models. By applying graph convolution on multichannel EEG features using the dynamic adjacency matrix, more discriminative features can be extracted for emotion classification.

Temporal attention is designed to dynamically capture the correlation between emotional EEG signals in the time domain. The temporal attention mechanism is defined as follows:


(12) $$\begin{array}{l} T=V\cdot \sigma \left(W_{3}X_{h}W_{4}+b_{t}\right) \end{array}$$

(13) $$\begin{array}{l} \hat{T}=\frac{T-E\left[T\right]}{\sqrt{\mathrm{Var}\left[T\right]}} \end{array}$$

$W_{3}\in {\mathbb{R}}^{T_{r}}$ and $W_{4}\in {\mathbb{R}}^{C\times N}$ are learnable parameters, and σ denotes the tanh activation function. Having the temporal attention weight matrix, we tuned the input ***X*_*h*_** by the temporal attention:


(14) $$\begin{array}{l} \hat{X_{h}}=\hat{T}X_{h} \end{array}$$

We utilize temporal attention to focus on valuable temporal information in EEG-based emotion recognition. The purpose of time-domain attention is to uncover the temporal patterns in EEG signals and assign importance weights based on their intrinsic similarities. By combining spatial attention with temporal attention, the model can extract more discriminative features from EEG signals and enhance the accuracy of emotion recognition. We use $\hat{X_{h}}$ as the input to the graph attention network and ***A*** as the adjacency matrix of the graph data.

## 4. Experiment

### 4.1. Datasets

The SEED dataset is an EEG-based dataset collected in the BCMI lab of Shanghai Jiao Tong University, known as the SJTU Emotion EEG Dataset (Zheng and Lu, 2015). The dataset contains a total of 62 channels of EEG signals from 15 subjects for 15 experiments. The researchers prepared 15 movie clips of approximately 4 min, which were divided into 3 categories: negative, neutral, and positive. Positive movies are comedies that stimulate positive emotions such as happiness; negative movies are tragic movies that stimulate negative emotions such as sadness, and neutral movies are world heritage documentaries that do not stimulate positive or negative emotions. The subjects were asked to watch these movie clips and were given 45 s to self-evaluate and calm down after each clip was shown.

The SEED-IV dataset is also from the BCMI lab (Zheng et al., 2018). This dataset features 168 movie clips that serve as a repository for four emotions (happy, sad, fearful, and neutral). Forty-four participants (22 females, all college students) were recruited to assess their emotions while watching the movie clips using keywords from four discrete emotions (happy, sad, neutral, and fearful) and rating 10 points (from –5 to 5) on two dimensions: valence and arousal.

The DREAMER dataset is a commonly used emotion recognition dataset (Katsigiannis and Ramzan, 2017). Researchers had subjects watch edited movie clips to elicit emotions from subjects and recorded EEG data using a 14-channel EEG acquisition device. These film clips consist of selected scenes from various movies that have been demonstrated to elicit a diverse array of emotions (Gabert-Quillen et al., 2015). After each movie clip was played, the researchers classified the emotions based on the subjects' ratings using 3 dimensions: potency, arousal, and dominance. The dataset contained 2 clips each of 9 emotion-evoking movies of happiness, excitement, bliss, calmness, anger, disgust, fear, sadness, and surprise, for a total of 18 movie clips.

### 4.2. Experiment settings

The STGATE model is implemented by PyTorch 1.10. The hyperparameters were tuned to obtain the best performance on the validation datasets.

The cross-subject experiments are conducted. Since there were multiple subjects, the leave-one-subject-out (LOSO) cross-validation strategy was applied in the experiments. The EEG data of one subject were used as the validation dataset, while the data of the other subjects were used as the training dataset. We repeatedly performed ten rounds of cross-validation experiments and the average accuracy and standard deviation of the test set are adopted as the performance criteria. The experiments in this paper use the Adam optimizer to accelerate the training process of the model with a batch size of 16 and a learning rate of 0.00001 (Kingma and Ba, 2014). Additionally, we use the Dropout algorithm to suppress the overfitting phenomenon of the model. The drop rate is set to 0.3. During the training process, the training set is stopped when the training loss is lower than 0.15.

## 5. Results

### 5.1. Results of experiments

Tables 1, 2 summarize the experimental results in terms of the average EEG emotion recognition accuracies and standard deviations of the STGATE method. To validate the effectiveness of our proposed method for EEG emotion recognition, we compared it with various machine learning and deep learning methods. Conventional classifiers such as supported vector machine (SVM) (Zhong et al., 2020) and transductive SVM (T-SVM) (Collobert et al., 2006) can be applied for cross-subject emotion recognition problems. Domain adaptation methods such as Transfer Component Analysis (TCA) (Pan et al., 2010) can also handle cross-subject emotion recognition problems. Pretrained Convolutional Neural Network (CNN) architectures have also been used in emotion recognition tasks (Cimtay and Ekmekcioglu, 2020). Rahman et al. proposed a method that hybridizes Principal Component Analysis (PCA) and t-statistics for feature extraction, and Artificial Neural Network (ANN) is applied for classification (Rahman et al., 2020). To deal with the domain shift problem between different subjects, a deep domain adaptation network (DAN) was proposed for cross-subject EEG signal recognition (Li et al., 2018). Likewise, to model asymmetric differences between two hemispheres of the EEG signal, a novel bi-hemispheric discrepancy model (BiHDM) was proposed for EEG emotion recognition (Li et al., 2020). He et al. explored the feasibility of combining Temporal Convolutional Networks (TCNs) and Adversarial Discriminative Domain Adaptation (ADDA) algorithms to solve the domain shift problem in EEG-based cross-subject emotion recognition (He et al., 2022). The Dynamical Graph Convolutional Neural Network (DGCNN) is a novel EEG-based emotion recognition model in which graph spectral convolution operation with dynamical adjacent matrix is applied (Song et al., 2018). Lew et al. propose a Regionally-Operated Domain Adversarial Network (RODAN) incorporate the attention mechanism to enable cross-domain learning to capture both spatial-temporal relationships among the EEG electrodes and an adversarial mechanism to reduce the domain shift in EEG signals (Lew et al., 2020).

**Table 1:** Leave-one-subject-out emotion recognition (accuracy/standard deviation) on the SEED and SEED-IV datasets.

**Model**	**SEED**	**SEED-IV**
SVM (Zhong et al., 2020)	56.73/16.29	37.99/12.52
T-SVM (Collobert et al., 2006)	72.53/14.00	(-)/(-)
TCA (Pan et al., 2010)	63.64/14.88	56.56/13.77
CNN (Cimtay and Ekmekcioglu, 2020)	78.34/**6.11**	(-)/(-)
PCA+ANN (Rahman et al., 2020)	84.3/(-)	(-)/(-)
RODAN (Lew et al., 2020)	(-)/(-)	60.75/(-)
DAN (Li et al., 2018)	83.81/8.56	58.87/8.13
BiHDM (Li et al., 2020)	85.40/7.53	69.03/8.66
DGCNN (Song et al., 2018)	79.95/9.02	(-)/(-)
GCN (Kipf and Welling, 2016)	84.95/7.51	72.23/**4.01**
GAT (Veličković et al., 2017)	85.56/6.75	70.33/4.57
**STGATE (Ours)**	**90.37**/6.18	**76.43**/5.01

The bold values indicate the maximum value of the current column in tables.

**Table 2:** Leave-one-subject-out emotion recognition (accuracy/standard deviation) on the DREAMER dataset.

**Model**	**Valence**	**Arousal**	**Average**
SVM (Zheng and Lu, 2015)	56.57/(-)	58.91/(-)	57.74/(-)
DBN (Zheng and Lu, 2015)	56.43/(-)	58.94/(-)	57.685/(-)
DGCNN (Song et al., 2018)	59.64/(-)	62.91/(-)	61.28/(-)
ADDA-TCN (He et al., 2022)	66.56/10.04	63.69/**6.57**	65.13/**8.31**
GCN (Kipf and Welling, 2016)	76.43/10.13	74.01/10.76	75.22/10.45
GAT (Veličković et al., 2017)	75.70/9.64	**75.31**/10.71	75.51/10.18
**STGATE (Ours)**	**77.44**/8.40	75.26/9.71	**76.35**/8.40

The bold values indicate the maximum value of the current column in tables.

Machine learning methods such as supported vector machine (SVM) and transfer component analysis (TCA) can be applied to address cross-subject emotion recognition problems. According to the experimental results in the SEED dataset and SEED-IV dataset, both machine learning methods, SVM, T-SVM and TCA, give lower accuracy than deep learning models. The performance of many deep learning methods, such as CNN, PCA+ANN, RODAN, DGCNN, DAN, and BiHDM, are better than that of the traditional machine learning methods (SVM and TCA), indicating that machine learning has difficulty obtaining valid features.

The proposed method achieves the highest accuracy in SEED, SEED-IV and DREAMER dataset. STGATE achieve 90.37% in SEED dataset and 76.43% in SEED-IV dataset. STGATE achieves 77.44% in the valence dimension, 75.26% in the arousal dimension, and 76.35% in the average value in both dimensions in DREAMER dataset, because the proposed STGATE can extract more useful information in the temporal and spatial dimensions. The proposed method treats EEG signals as non-Euclidean data and uses graph representations and attention mechanisms to extract the spatial and temporal characteristics of EEGs. STGATE compensates for the limitations of convolutional neural networks and can handle the feature extraction problem of non-Euclidean data with topological graph structure. The combination of the transformer encoder and STGAT enhances the performance of the network. The modeled graph representations restore the spatial and temporal connectivity of the data and make STGATE extract more discriminative emotional features that can be used to accurately classify and identify the emotional states of subjects. The proposed STGATE extracts electrode-level information through TLB and spatial features based on an adaptive graph structure. Therefore, we can see that our proposed method achieves the best accuracy results on the SEED, SEED-IV, and DREAMER datasets.

### 5.2. Ablation study

To verify the effectiveness of each module in STGATE, we removed them one at a time or replaced some of the layers and evaluated the performance of the ablated model. As shown in Table 3, we trained several models to verify the impact of the modules in STGATE. For the baseline model, we only deployed a series of multi-kernel 2D convolutions during training and testing. "TGAT" refers to using spatial-temporal graph attention without spatial attention and is similar to “SGAT.” The TLB was utilized to attentively fuse the node-level features, and the convolutional layers downsampled the multi-channel EEG input and learned the time-frequency representations. The transformer encoder enhanced the long-range dependency capturing ability. According to the results shown in Table 3, removing the TLB module caused the accuracy to drop from 90.27% to 87.88%, a decrease of 2.39%. When we removed the transformer encoder in the TLB, the accuracy dropped from 86.2% to 83.86%, a decrease of 2.36%. The results show the effectiveness of the TLB module.

**Table 3:** Performance of our proposed modules on the SEED and SEED-IV datasets.

**Model**	**SEED**	**SEED-IV**
Baseline	83.86/9.88	71.17/4.48
TLB	86.22/8.57	73.98/4.12
SGAT	86.67/6.98	75.39/5.49
SGAT+TLB	88.56/5.91	75.85/5.06
TGAT	86.84/7.40	76.04/4.76
TGAT+TLB	89.30/**5.69**	76.28/**4.06**
SGAT+TGAT (STGAT)	87.88/7.38	75.52/4.30
**SGAT+TGAT+TLB (STGATE)**	**90.37**/6.18	**76.43**/5.01

The bold values indicate the maximum value of the current column in tables.

The STGAT module aimed to learn the dynamic spatial-temporal representations of the graph. Spatial attention built an adaptive adjacency matrix through several learnable parameters, making the graph structure dynamically change during the training process. The dynamic adjacency matrix had the potential to extract informative correlations among electrodes. Temporal attention was similar to spatial attention and captured temporal information through several learnable parameters. As shown in Table 3, removing the STGAT module caused the accuracy to drop from 90.27% to 86.22%, a decrease of 4.05%. When we removed the spatial and temporal attention in STGAT, the accuracy dropped from 87.88% to 86.84%, decreasing by 1.04%, and 87.88% to 86.67%, decreasing by 1.21%, respectively.

The performance of the STGATE model outperforms that of other models by a significant margin. This is attributed to the ability of the spatial and temporal attention modules to capture potential EEG signal features, while the transformer encoder helps to enhance long-range dependency capturing ability. The models with STGAT and TLB modules significantly outperform those without these modules. The transformer learning block aggregates time-frequency features using convolution and a transformer encoder, while the spatial-temporal graph attention captures inter-channel connections *via* an adaptive adjacency matrix and temporal information using temporal attention. Therefore, both the transformer learning block and the dynamic graph convolution are essential components of the STGATE model.

### 5.3. The impact of feature selection

**Figure 2:**
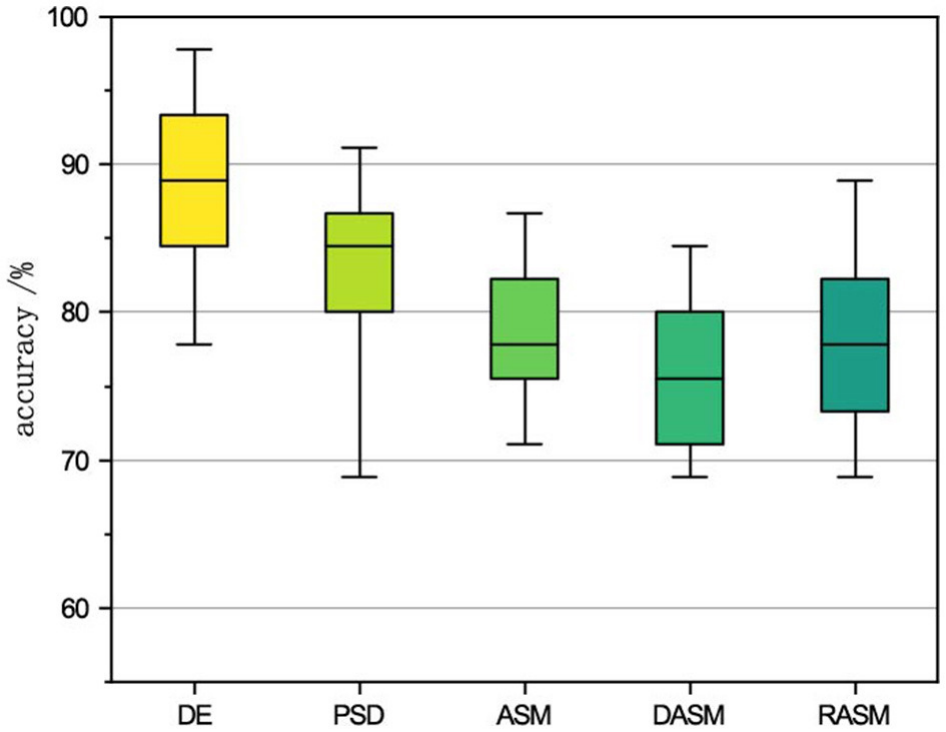
The impact of different features on SEED accuracy.

Raw emotional EEG is a non-linear random signal with a large amount of data redundancy and a low signal-to-noise ratio (Balasubramanian et al., 2018). The EEG signal features, such as power spectral density (PSD) and differential entropy, are more representative of the prominent features of the EEG signal in certain aspects. Therefore, the emotion classification task generally uses the feature of the EEG signal for classification. Therefore, we study the impact of the feature selection of STGATE on classification performance. Figure 2 shows the emotion recognition accuracies and standard deviations of the proposed STGATE model with different features, including DE, PSD, ASM, DASM, and RASM features, in the SEED dataset. The DE feature is the most discriminated feature, while the performance of other features is much lower. The DE feature obtained the highest classification accuracy (90.37%) and lowest standard deviation, followed by PSD (83.17%). RASM (rational asymmetry), DASM (differential asymmetry), and ASM (asymmetry) are calculated from DE features designed to express asymmetry (Shi et al., 2013). The average accuracies of the RASM, DASM, and ASM features are close to each other, 78.07%, 75.76%, and 77.75%, respectively. The result implies that the DE feature is more suitable for EEG emotion recognition than the traditional feature.

### 5.4. Visualization

**Figure 3:**
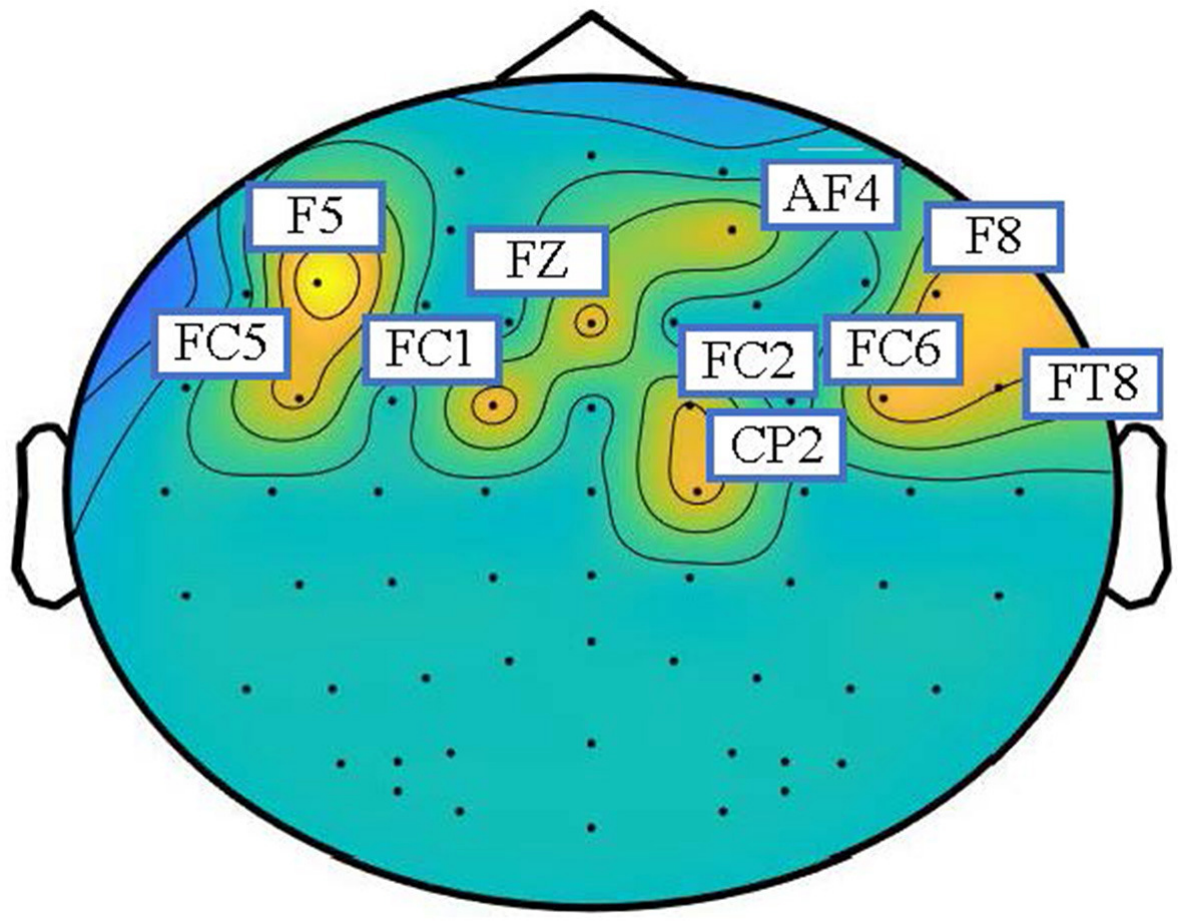
Topographic map of adjacency matrices on SEED datasets. The weighted electrodes are mainly distributed in the frontal and parietal lobes.

As shown in Figure 3, the topographic map is utilized to analyze the inter-channel connections of the learned graphs for emotion recognition in the SEED dataset. The adjacent matrices are extracted at the end of training and transformed into a topographic map. To better show which part of the connections is more informative, we extracted the adjacency matrices of the EEG samples of all the subjects, averaged all the matrices, took the largest ten values, and set the others to zero. The topographic map of the adjacency matrices is shown in Figure 3. According to the topographic map, the frontal lobe plays an important role in the classification of emotions. The F5, FC5, FC1, FZ, F8, AF8, CP2, FC6, FT8, and C2 channels have more weight than other channels, which means that these channels provide more information during the training process. According to previous studies, the pre-frontal, parietal and occipital channels may be the most associated with emotions (Zheng and Lu, 2015; Zhong et al., 2020; Ding et al., 2021). The visualization results basically coincide with the observations in neuroscience. Therefore, the topographic map indicates that the dynamic adjacency matrix gives more weight to the emotionally relevant EEG channels to enhance the potential ability of STGATE.

## 6. Conclusion

In this paper, we proposed STGATE, a novel method for EEG-based emotion recognition that can dynamically learn the inter-channel relationships of EEG emotion signals. The STGATE is composed of two modules, TLB and STGAT. The TLB module employs 2D convolutions and a transformer encoder to downsample EEG signals and capture long-range information. The STGAT module dynamically captures correlations between EEG electrodes in the spatial domain and temporal EEG information using a time-spatial attention mechanism. The experimental results demonstrate that STGATE achieves higher classification accuracies compared to existing methods for cross-subject EEG-based emotion recognition. However, a limitation of this study is the small sample size of the publicly available dataset used in the article and the lack of sufficient reliable data within the dataset. Nevertheless, our proposed method has the potential to inspire new methodologies for emotion recognition and affective computing.

## Data availability statement

The original contributions presented in the study are included in the article/supplementary material, further inquiries can be directed to the corresponding author.

## Author contributions

JL proposed the idea and wrote the manuscript. WP conducted the experiments. HH and JP provided advice on the research approaches, signal processing, and checked and revised the manuscript. FW offered important help on guided the experiments and analysis methods. All authors contributed to the article and approved the submitted version.
